# Thermally modulated resonant quantum transport in asymmetric nanoscale junctions for optimal bias and power generation analysis

**DOI:** 10.1038/s41598-026-58972-3

**Published:** 2026-06-25

**Authors:** Arafa H. Aly

**Affiliations:** TH-PPM Group, Physics Department, Faculty of Sciences, Beni-Suef University, Beni Suef, 62111 Egypt

**Keywords:** Asymmetric resonant transport, Heat current modulation, Thermal transport, Resonant energy level, Bias window, Structural asymmetry, Finite-bias activation, Phenomenological model, Engineering, Materials science, Nanoscience and technology, Physics

## Abstract

A phenomenological effective transport model inspired by Landauer-type resonant transport concepts is presented to investigate thermally modulated transport behavior in an asymmetric nanoscale junction under combined electrical and thermal driving. The proposed framework incorporates resonance alignment, thermal resonance modulation, finite-bias activation, damping effects, and structural asymmetry within a computationally efficient formulation. The transport response is systematically analyzed as functions of bias voltage, asymmetry strength, temperature, and resonance energy. The results show that increasing structural asymmetry reduces the magnitudes of the heat-current proxy, charge-current magnitude, and electrical power magnitude due to weaker effective transport coupling. However, the optimal bias voltage associated with the maximum electrical power magnitude remains only weakly affected within the investigated parameter range because the asymmetry factor primarily scales the transport amplitude while weakly modifying the resonance-alignment condition. The simulations further demonstrate that the maximum electrical power magnitude increases with temperature, whereas the thermal sensitivity gradually decreases at elevated temperatures. In addition, the optimal operating bias increases with resonance energy according to the resonance-alignment condition included in the model. A two-dimensional operating map identifies a stable high-performance transport region near 4–4.5 mV. All numerical parameters used in the simulations are explicitly reported to support reproducibility. The proposed framework provides a simplified and physically interpretable platform for analyzing resonance-dominated transport trends in asymmetric nanoscale systems and may serve as a useful basis for future microscopic or experimentally calibrated studies.

## Introduction

The continuous demand for efficient energy conversion and thermal management at the nanoscale has stimulated extensive research into thermoelectric and quantum transport systems. Nanoscale devices capable of controlling charge and heat flow have attracted considerable attention because of their potential applications in energy harvesting, sensing, and thermal regulation. At these length scales, transport behavior deviates substantially from classical conduction and becomes strongly governed by quantum effects, where electron transmission depends sensitively on energy filtering and resonance conditions^1–3^. The Landauer transport framework and its later extensions provide the theoretical basis for describing electronic transport in terms of energy-dependent transmission probabilities across nanoscale junctions^4–6^. One of the most effective approaches for improving thermoelectric transport behavior is the use of energy-selective transport through resonant structures. Mahan and Sofo showed that sharply peaked transmission functions can enhance thermoelectric performance through efficient energy filtering^7^. Resonant tunneling systems have therefore been widely investigated as model platforms for nanoscale transport, where the transmission probability can often be represented by a Lorentzian or Breit–Wigner-type resonance profile centered around a discrete energy level^8^. Such systems allow transport properties to be controlled through the alignment between the resonant level and the electrochemical transport window^9–11^. Molecular thermoelectricity and quantum-interference-enhanced transport have further emphasized the importance of transmission-shape engineering in nanoscale systems. Bergfield and Stafford demonstrated that coherent transport in single-molecule junctions can strongly enhance thermoelectric response near transmission nodes^12^. Bergfield, Solis, and Stafford later showed that higher-order transmission supernodes can produce strong thermoelectric enhancement and affect molecular-scale heat-engine performance^13^. Related studies by Lambert, Sadeghi, and Al-Galiby, as well as Sadeghi et al., highlighted the role of quantum and phonon interference in improving molecular-scale thermoelectric transport^14,15^.

The problem of optimal power generation and thermoelectric efficiency in nanoscale systems has also received significant attention. Whitney analyzed quantum bounds on thermoelectric efficiency and finite-power operation in Landauer-type systems^16,17^. Josefsson et al. further investigated optimal power and efficiency in single quantum-dot heat engines using both theoretical and experimental approaches^18^. These studies demonstrate that optimal operating conditions are closely linked to resonance structure, transmission characteristics, and non-equilibrium transport behavior.

Structural asymmetry is another important factor influencing nanoscale transport. In realistic nanoscale junctions, the coupling between the transport region and the surrounding reservoirs is rarely perfectly symmetric. Unequal coupling conditions can substantially modify charge transport, heat transport, and power characteristics by altering the effective transmission amplitude and resonance broadening^19–23^. Previous studies on nanoscale and molecular thermoelectric systems have also emphasized the coupled nature of heat and charge transport under non-equilibrium conditions and the role of transport asymmetry in modifying device performance^24–26^. Recent advances in low-dimensional materials, mesoscopic systems, and thermoelectric device engineering have further highlighted the importance of energy-level engineering, interfacial design, and simplified modeling approaches for understanding nanoscale energy conversion^27–33^. Although fully microscopic Landauer-based calculations provide rigorous descriptions of quantum transport, phenomenological and semi-empirical models can provide useful physical insight into dominant trends while remaining computationally efficient and reproducible. Despite these advances, several limitations still persist. Many reported approaches depend either on fully microscopic numerical calculations that provide limited physical interpretability or on simplified formulations that fail to simultaneously incorporate resonance alignment, thermal modulation, finite-bias activation, damping behavior, and structural asymmetry within a single unified framework. In addition, while an optimal operating bias is often observed in resonant transport systems, its dependence on resonance energy and asymmetry conditions remains insufficiently clarified in simplified transport formulations. In the present work, a phenomenological effective transport model inspired by Landauer-type resonant transport concepts is developed to investigate thermally modulated transport behavior in an asymmetric nanoscale junction under coupled electrical and thermal driving conditions. The proposed framework incorporates resonance alignment, thermal resonance modulation, finite-bias activation, damping effects, and structural asymmetry within a computationally efficient formulation. Unlike direct microscopic Landauer calculations, the present approach is intended to provide a simplified and physically interpretable description of the dominant transport trends governing the heat-current proxy, charge-current magnitude, electrical power magnitude, and optimal operating bias. The numerical analysis systematically examines the influence of bias voltage, asymmetry strength, temperature, and resonance energy on the transport response of the system. The results show that increasing structural asymmetry suppresses the transport magnitude due to weaker effective coupling, whereas the optimal operating bias remains only weakly affected within the investigated parameter range because the asymmetry factor primarily scales the transport amplitude while weakly modifying the resonance-alignment condition. Overall, the proposed framework provides a reproducible and physically interpretable platform for analyzing resonance-dominated transport trends in asymmetric nanoscale systems and may serve as a useful basis for future microscopic or experimentally calibrated investigations.

## Theoretical background and performance metrics

A thermally driven asymmetric nanoscale resonant junction is considered, in which two metallic reservoirs are coupled to a central resonant region through unequal tunnelling barriers. The left and right reservoirs are maintained at temperatures ${T}_{L}$ and ${T}_{R}$, respectively, while an external bias voltage ${V}_{b}$ is applied across the junction. The transport response is assumed to be dominated by a single effective resonant level whose position is thermally shifted and whose alignment with the bias-defined transport window controls the magnitude of charge and heat transport.

The physical motivation of the model follows the energy-filtering picture of resonant quantum transport. In a microscopic Landauer formulation, the charge current through a coherent two-terminal junction is commonly written as^1–6^

1 $$I=\frac{2q}{h}{\int}_{-\infty }^{+\infty }\mathcal{T}\left(E\right)\left[{f}_{L}\left(E\right)-{f}_{R}\left(E\right)\right]dE$$

where $I$ is the charge current, $q$ is the elementary charge, $h$ is Planck’s constant, $E$ is the carrier energy, $\mathcal{T}\left(E\right)$ is the energy-dependent transmission probability, and ${f}_{L}\left(E\right)$ and ${f}_{R}\left(E\right)$ are the Fermi–Dirac distribution functions of the left and right reservoirs, respectively.

The heat current associated with reservoir $\alpha$ may be expressed as

2 $${J}_{\alpha }=\frac{2}{h}{\int}_{-\infty }^{+\infty }\left(E-{\mu}_{\alpha }\right)\mathcal{T}\left(E\right)\left[{f}_{L}\left(E\right)-{f}_{R}\left(E\right)\right]dE$$

where ${J}_{\alpha }$ is the heat current measured relative to reservoir $\alpha$, and ${\mu}_{\alpha }$ is the chemical potential of that reservoir. Equations (1) and (2) are introduced as the physical background of resonant energy filtering. The present work, however, does not numerically evaluate the full Landauer integrals. Instead, it adopts a compact phenomenological effective model that preserves the dominant effects required for the present analysis: resonance alignment, thermal modulation, asymmetric coupling, finite-bias activation, and high-bias damping. Therefore, the calculated currents should be interpreted as effective transport quantities rather than fully microscopic Landauer currents.

For a single resonant transport channel, the transmission is represented by an effective Lorentzian profile^1–6^,

3 $${\mathcal{T}}_{\mathrm{e}\mathrm{f}\mathrm{f}}\left({V}_{b},{T}_{L},{T}_{R}\right)=\frac{{\Gamma}_{\mathrm{t}\mathrm{o}\mathrm{t}}^{2}}{\Delta {E}^{2}+{\Gamma}_{\mathrm{t}\mathrm{o}\mathrm{t}}^{2}}$$

where ${\mathcal{T}}_{\mathrm{e}\mathrm{f}\mathrm{f}}$ is the effective dimensionless transmission factor, $\Delta E$ is the effective detuning between the resonant level and the bias-induced transport window, and ${\Gamma}_{\mathrm{t}\mathrm{o}\mathrm{t}}$ is the total resonance broadening. The total broadening is defined as

4 $${\Gamma}_{\mathrm{t}\mathrm{o}\mathrm{t}}={\Gamma}_{L}+{\Gamma}_{R}$$

where ${\Gamma}_{L}$ and ${\Gamma}_{R}$ are the effective coupling-induced broadenings associated with the left and right barriers, respectively. In the MATLAB implementation, these broadenings are expressed in meV.

The resonance detuning is written as

5 $$\Delta E={E}_{r}-{\beta}_{V}{V}_{b}$$

where ${E}_{r}$ is the thermally shifted resonance energy, ${V}_{b}$ is the applied bias voltage, and ${\beta}_{V}$ is the phenomenological bias-alignment coefficient with units of meV/mV. This term describes the shift of the effective transport window under applied bias.

Thermal modulation is introduced by allowing the resonant level to shift linearly with the imposed temperature difference,

6 $${E}_{r}={E}_{r0}+{\alpha}_{T}\left({T}_{L}-{T}_{R}\right)$$

where ${E}_{r0}$ is the base resonance energy, ${\alpha}_{T}$ is the thermal resonance-shift coefficient in meV/K, and ${T}_{L}-{T}_{R}$ is the applied temperature difference. This relation represents the effective thermal displacement of the resonant state.

Structural asymmetry is introduced through the dimensionless barrier parameters ${Z}_{L}$ and ${Z}_{R}$. In the present effective model, asymmetry mainly scales the transport amplitude through

7 $${A}_{\mathrm{a}\mathrm{s}\mathrm{y}\mathrm{m}}=\frac{1}{1+{a}_{L}{Z}_{L}+{a}_{R}{Z}_{R}}$$

where ${A}_{\mathrm{a}\mathrm{s}\mathrm{y}\mathrm{m}}$ is the dimensionless asymmetry factor, and ${a}_{L}$ and ${a}_{R}$ are empirical weighting coefficients describing the relative influence of the left and right barriers. This formulation reduces the transport magnitude as the effective barrier asymmetry increases.

To include the weak effect of asymmetry on resonance linewidth, the left and right broadenings are defined as

8 $${\Gamma}_{L}={\Gamma}_{L0}\left(1-{\lambda}_{L}{Z}_{L}\right)$$

and

9 $${\Gamma}_{R}={\Gamma}_{R0}\left(1-{\lambda}_{R}{Z}_{R}\right)$$

where ${\Gamma}_{L0}$ and ${\Gamma}_{R0}$ are the reference left and right broadenings, while ${\lambda}_{L}$ and ${\lambda}_{R}$ are dimensionless broadening-reduction coefficients. These terms allow the resonance linewidth to respond weakly to barrier variations while preserving the dominant resonance-alignment mechanism.

The low-bias transport onset is described using the smooth activation function

10 $${F}_{\mathrm{o}\mathrm{n}}=1-\mathrm{e}\mathrm{x}\mathrm{p}\left[-{\left(\frac{{V}_{b}}{{V}_{\mathrm{t}\mathrm{u}\mathrm{r}\mathrm{n}}}\right)}^{2}\right]$$

where ${F}_{\mathrm{o}\mathrm{n}}$ is a dimensionless turn-on function and ${V}_{\mathrm{t}\mathrm{u}\mathrm{r}\mathrm{n}}$ is the characteristic turn-on voltage. This function suppresses unphysical transport at the zero-bias limit.

The finite-bias suppression is represented by

11 $${F}_{\mathrm{d}\mathrm{e}\mathrm{c}\mathrm{a}\mathrm{y}}=\mathrm{e}\mathrm{x}\mathrm{p}\left(-\frac{{V}_{b}}{{V}_{\mathrm{d}\mathrm{e}\mathrm{c}\mathrm{a}\mathrm{y}}}\right)$$

where ${F}_{\mathrm{d}\mathrm{e}\mathrm{c}\mathrm{a}\mathrm{y}}$ is a dimensionless damping factor and ${V}_{\mathrm{d}\mathrm{e}\mathrm{c}\mathrm{a}\mathrm{y}}$ is the characteristic voltage scale associated with high-bias decay. This term phenomenologically represents non-ideal scattering, decoherence, and transport suppression at larger bias.

Using the above effective factors, the charge-current magnitude calculated in the simulations is

12 $$\left|I\right|={A}_{I}{A}_{\mathrm{a}\mathrm{s}\mathrm{y}\mathrm{m}}{V}_{b}{F}_{\mathrm{o}\mathrm{n}}{F}_{\mathrm{d}\mathrm{e}\mathrm{c}\mathrm{a}\mathrm{y}}\left({c}_{1}+{c}_{2}{\mathcal{T}}_{\mathrm{e}\mathrm{f}\mathrm{f}}\right)\left[1+\chi \left({T}_{L}-{T}_{R}\right)\right]$$

where $\left|I\right|$ is the effective charge-current magnitude, ${A}_{I}$ is the current scaling coefficient, ${c}_{1}$ and ${c}_{2}$ weight the non-resonant and resonant contributions, respectively, and $\chi$ is the weak thermal enhancement coefficient. The voltage ${V}_{b}$ is used in mV inside the current expression, consistent with the MATLAB implementation, while conversion to volts is applied when calculating electrical power.

The heat-current response is treated as an effective heat-current proxy rather than a reservoir-resolved microscopic heat current. It is written as

13 $$J=-{A}_{J}{A}_{\mathrm{a}\mathrm{s}\mathrm{y}\mathrm{m}}\left(\frac{{T}_{L}-{T}_{R}}{{T}_{L}-{T}_{R}+{\delta}_{T}}\right){\mathcal{T}}_{\mathrm{e}\mathrm{f}\mathrm{f}}{F}_{\mathrm{o}\mathrm{n}}\left({d}_{1}+{d}_{2}{F}_{\mathrm{d}\mathrm{e}\mathrm{c}\mathrm{a}\mathrm{y}}\right)$$

where $J$ is the effective heat-current proxy, ${A}_{J}$ is the heat-current scaling coefficient, ${\delta}_{T}$ is a thermal saturation parameter, and ${d}_{1}$ and ${d}_{2}$ are dimensionless weighting constants. The negative sign denotes the chosen direction of heat flow under the imposed temperature gradient.

The electrical power magnitude is evaluated as

14 $$\left|P\right|=\left|I\right|{V}_{b}^{\left(\mathrm{V}\right)}$$

where $\left|P\right|$ is the electrical power magnitude and ${V}_{b}^{\left(\mathrm{V}\right)}$ is the applied bias expressed in volts. This definition represents the magnitude of electrical power associated with the transport current and should not be interpreted as a rigorous thermodynamic generated power unless a complete sign convention and heat-engine operating regime are explicitly imposed.

To avoid ambiguity with thermodynamic efficiency, the previous efficiency-like metric is replaced by a dimensionless performance indicator,

15 $$\mathrm{D}\mathrm{P}\mathrm{I}={\eta}_{0}\frac{\left|P\right|}{\left|J\right|+\varepsilon }$$

where DPI is a dimensionless comparative performance indicator, ${\eta}_{0}$ is a normalization coefficient, and $\varepsilon$ is a small numerical stabilizing quantity introduced to avoid division by zero. DPI is used only for relative comparison between operating conditions and does not represent thermodynamic efficiency.

The thermal sensitivity is obtained from the variation of the maximum electrical power magnitude with left-reservoir temperature,

16 $${S}_{T}=\frac{d{P}_{\mathrm{m}\mathrm{a}\mathrm{x}}}{d{T}_{L}}$$

where ${S}_{T}$ is the thermal sensitivity in W/K and ${P}_{\mathrm{m}\mathrm{a}\mathrm{x}}$ is the maximum value of $\left|P\right|$ extracted from the bias-voltage sweep at each value of ${T}_{L}$.

The numerical procedure consists of sweeping the bias voltage over the range 0–5 mV for different asymmetry configurations, reservoir temperatures, and resonance energies. For each operating condition, ${\mathcal{T}}_{\mathrm{e}\mathrm{f}\mathrm{f}}$, $\left|I\right|$, $J$, $\left|P\right|$, and DPI are evaluated. The optimal bias voltage ${V}_{\mathrm{o}\mathrm{p}\mathrm{t}}$ is then extracted as the bias corresponding to the maximum electrical power magnitude. Within the assumptions of the present phenomenological model, the weak dependence of ${V}_{\mathrm{o}\mathrm{p}\mathrm{t}}$ on asymmetry arises because ${A}_{\mathrm{a}\mathrm{s}\mathrm{y}\mathrm{m}}$ mainly scales the transport amplitude, while the resonance condition in Eq. (5) primarily determines the position of the power maximum.

## Results and discussion

**Fig. 1:**
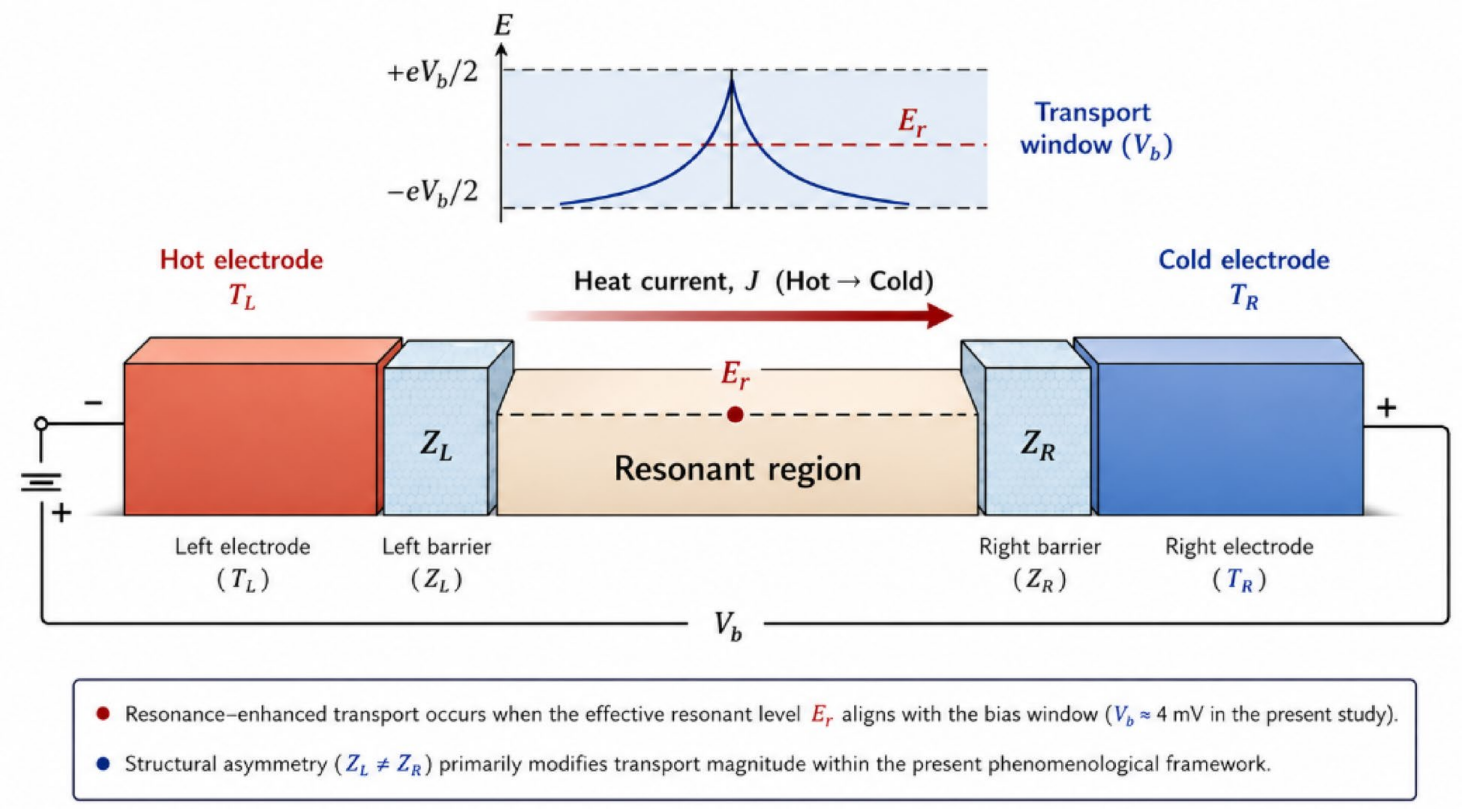
Schematic illustration of the proposed asymmetric resonant transport junction under coupled electrical and thermal driving.

As illustrated in Fig. 1, the proposed asymmetric resonant transport system consists of two electrodes maintained at different temperatures ($T_{L} \;{\mathrm{and}}\;T_{R}$), separated by asymmetric barriers (${Z}_{L}\ne {Z}_{R}$) and a central resonant region characterized by the effective resonant level ${E}_{r}$. When ${E}_{r}$ aligns with the bias-induced transport window, resonance-enhanced heat transport occurs from the hot electrode toward the cold electrode.

The structural asymmetry mainly influences the transport magnitude, whereas the resonance alignment condition governs the activation of the enhanced heat-current response. Based on this physical framework, the following results investigate the effects of bias conditions, resonance evolution, and barrier asymmetry on the thermal transport characteristics of the proposed structure.

The transport characteristics of the proposed thermally modulated asymmetric resonant junction were systematically investigated as functions of bias voltage, structural asymmetry, temperature, and resonance energy. The numerical analysis was performed using the phenomenological effective transport framework introduced in the previous section. The results reveal a strongly resonance-dominated transport regime, where both the charge-current magnitude and the heat-current proxy are primarily governed by the alignment between the effective resonance energy and the bias-defined transport window.

**Fig. 2:**
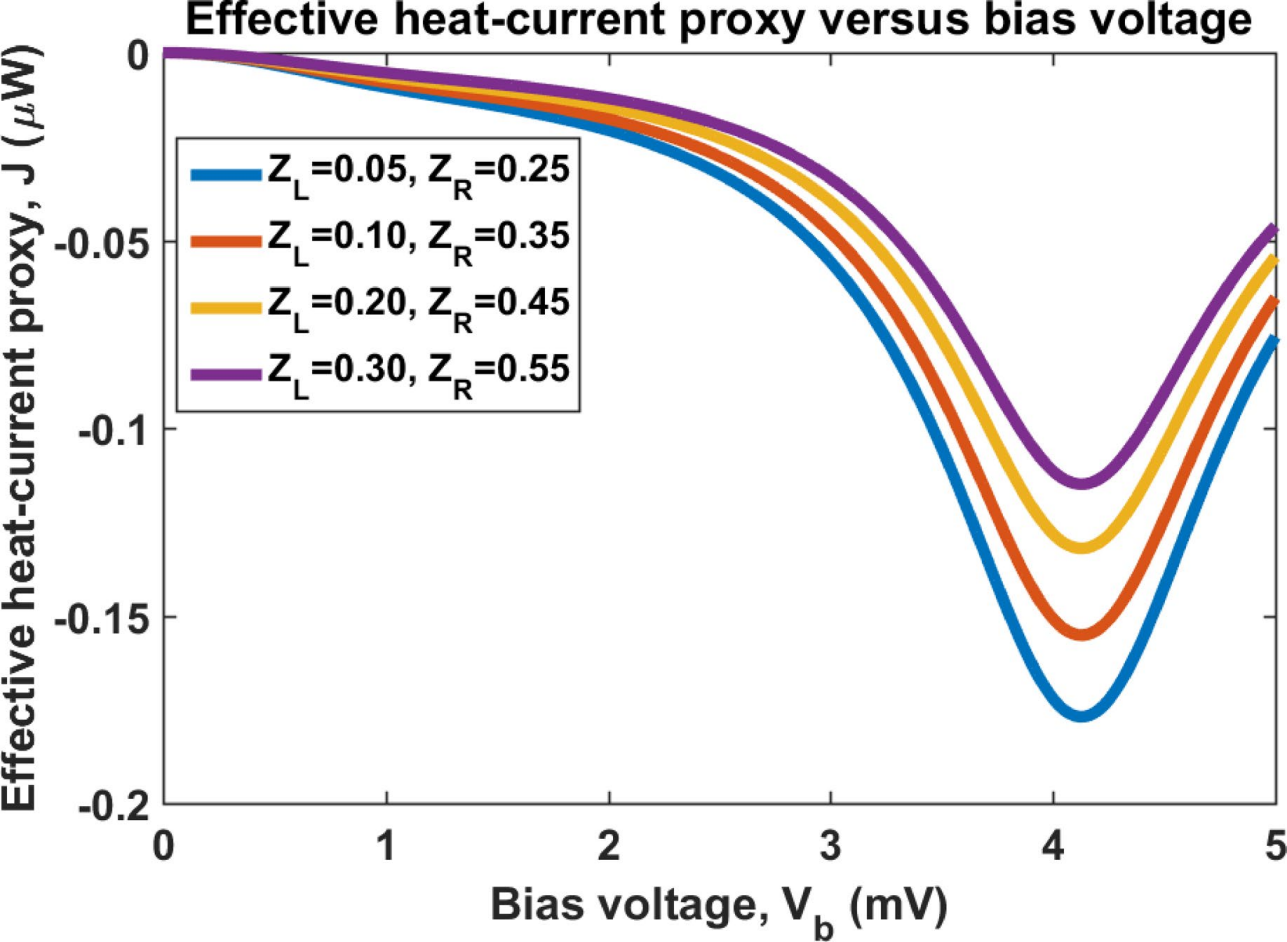
Heat-current proxy versus bias voltage for different asymmetry configurations.

The variation of the effective heat-current proxy with bias voltage for different asymmetry configurations is shown in Fig. 2.

**Fig. 3:**
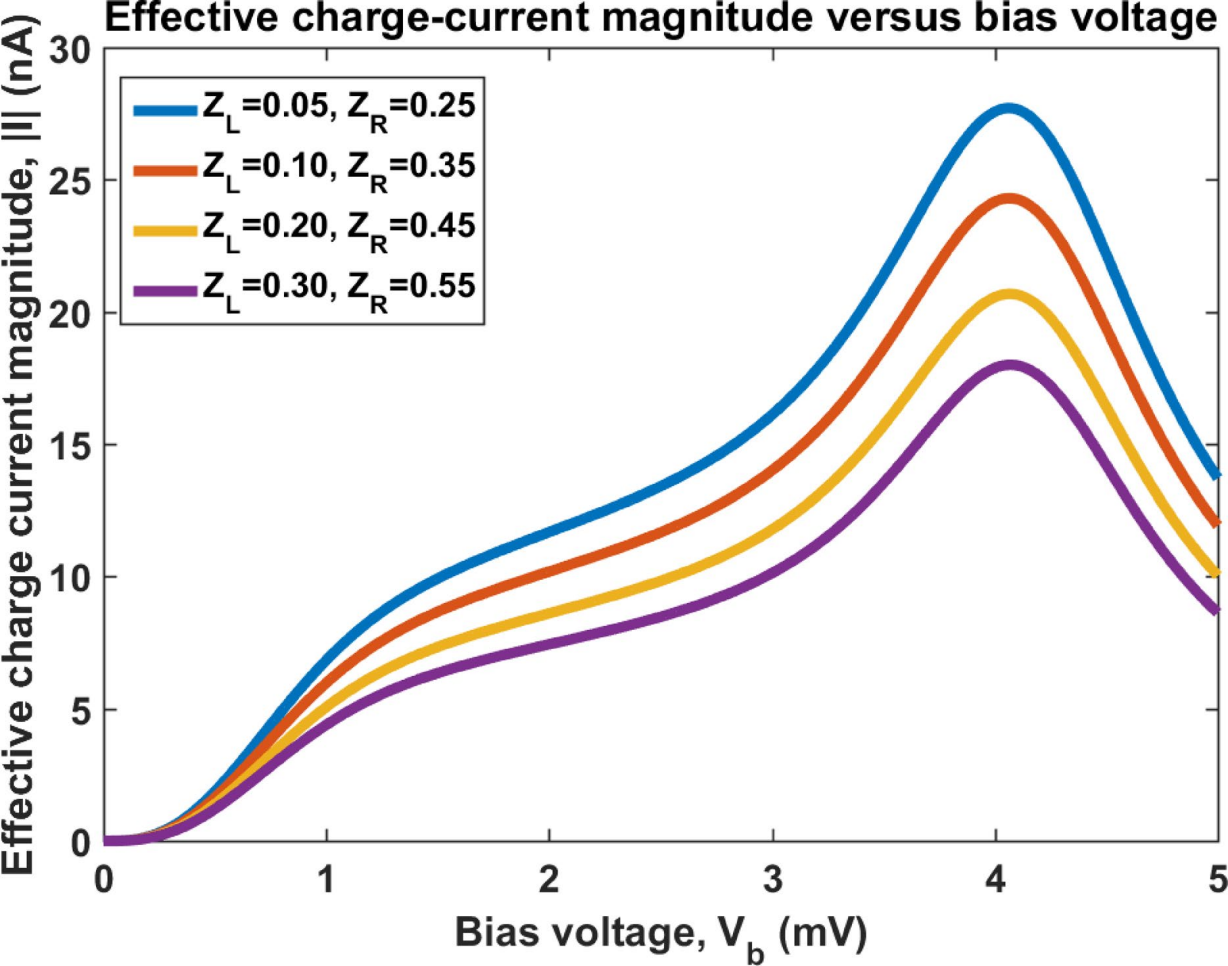
Charge-current magnitude versus bias voltage for different asymmetry configurations.

The heat-current magnitude initially increases with increasing bias voltage before gradually approaching a saturation-like regime at higher bias values. This behavior originates from the combined influence of resonance-assisted transport and the finite-bias activation mechanism included in the phenomenological model. Increasing the asymmetry parameters ZL and ZR reduces the overall transport magnitude due to the reduction of the effective coupling through the asymmetry factor. However, the general shape of the transport curves remains nearly unchanged, indicating that resonance alignment remains the dominant factor controlling the transport condition. The calculated charge-current magnitude is presented in Fig. 3.

**Fig. 4:**
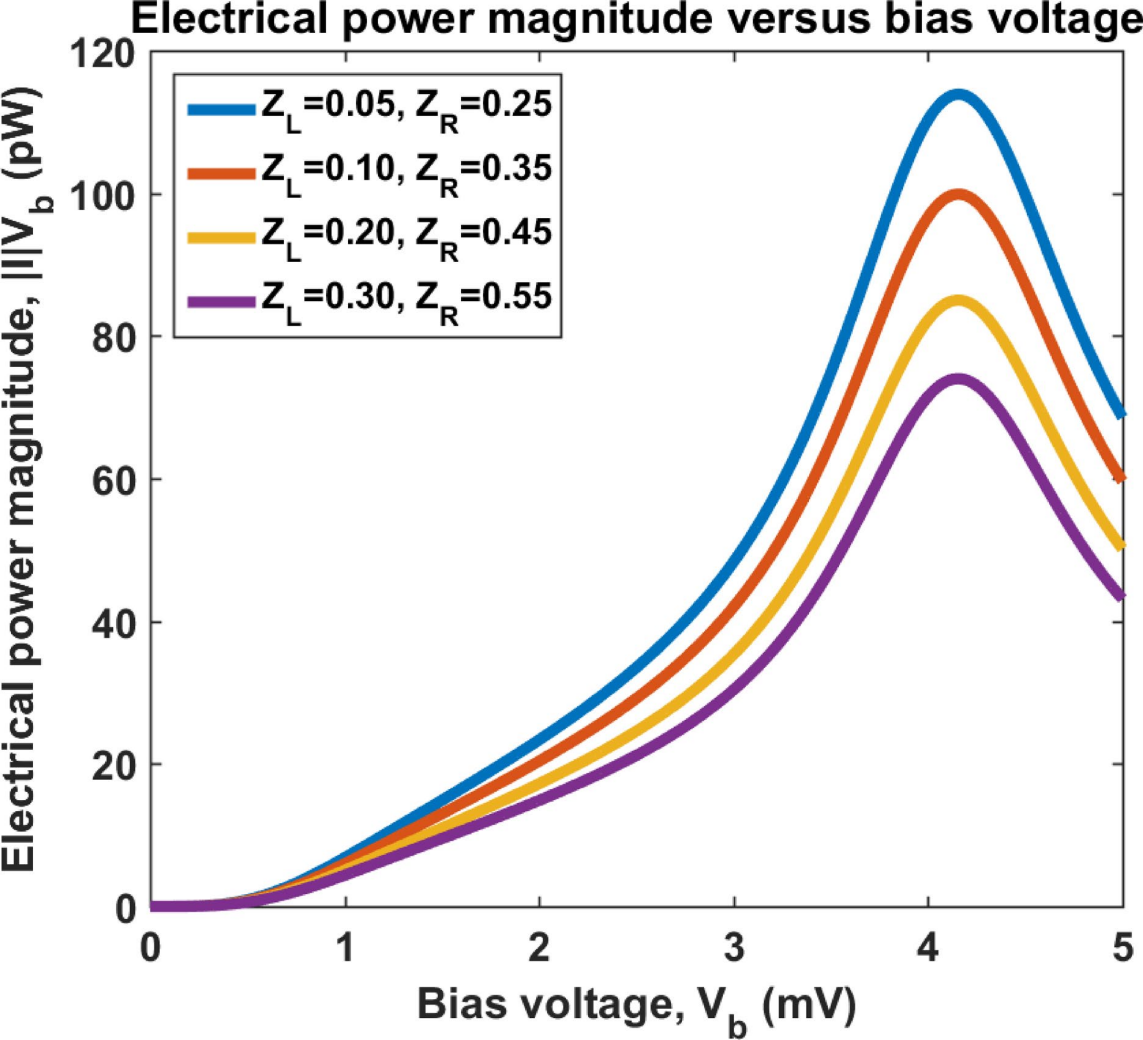
Electrical power magnitude versus bias voltage, showing a stable optimum near 4–4.5 mV.

Unlike the heat-current response, the charge-current magnitude exhibits a pronounced maximum at finite bias voltage. At low bias values, transport remains suppressed by the smooth turn-on function. As the bias voltage increases, the resonance alignment improves and the transport magnitude rises rapidly. Beyond the optimal region, the damping function progressively suppresses transport, leading to a decrease in the current magnitude. The results further demonstrate that increasing asymmetry reduces the current amplitude while producing only weak changes in the position of the transport maximum. The electrical power magnitude as a function of bias voltage is shown in Fig. 4.

A distinct maximum appears near V_b_ ≈ 4–4.5 mV for all investigated asymmetry configurations. The existence of this stable operating region confirms the resonance-dominated nature of the transport process. While increasing asymmetry reduces the power magnitude, the optimal bias voltage remains nearly unchanged. Within the assumptions of the present phenomenological framework, this behavior arises because the asymmetry factor primarily scales the transport amplitude while only weakly modifying the resonance-alignment condition itself. Consequently, the weak dependence of the optimal bias on asymmetry should be interpreted as a characteristic feature of the present effective transport model rather than a universal prediction for all resonant nanoscale systems.

**Fig. 5:**
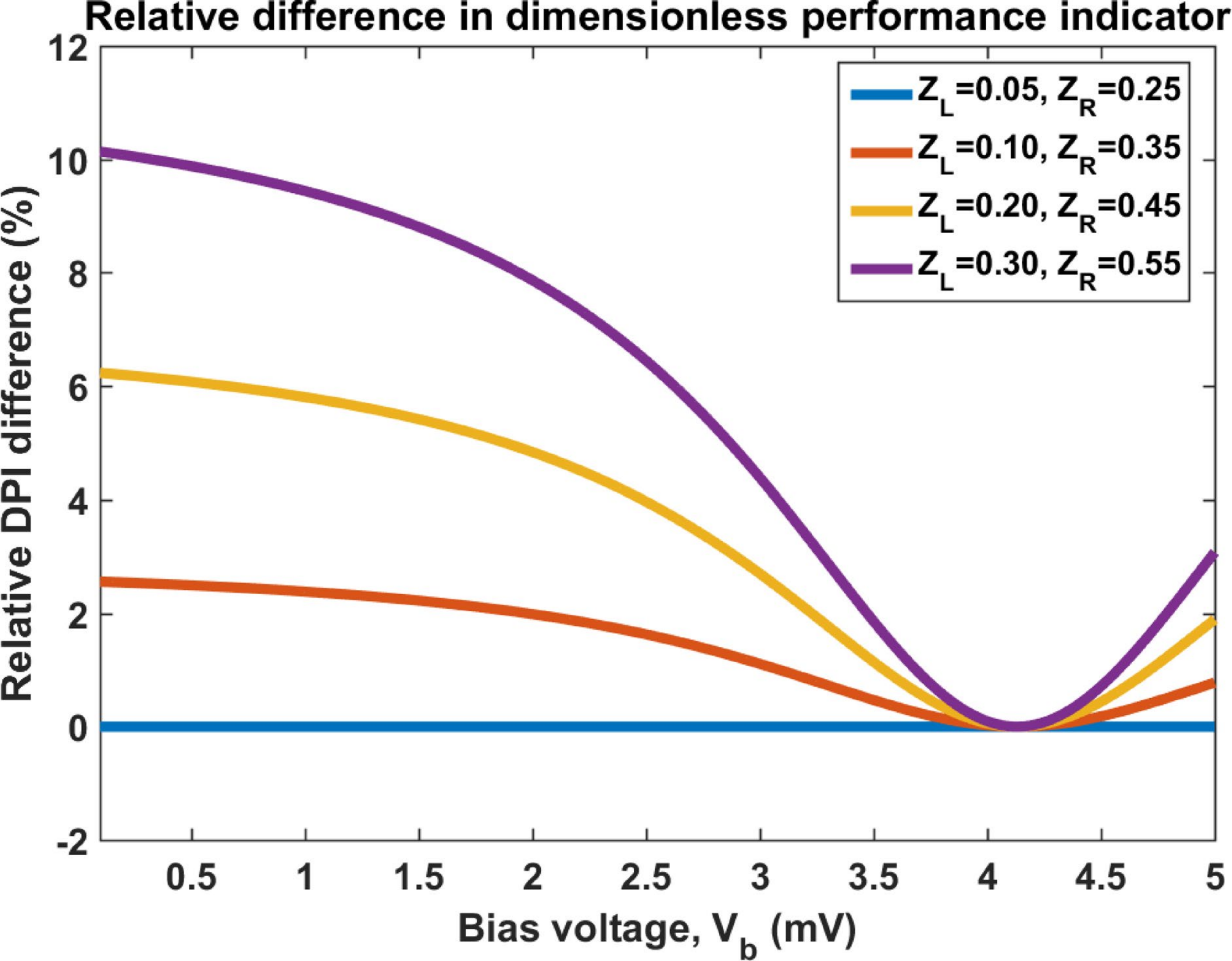
Relative variation of the dimensionless performance indicator (DPI) with bias voltage.

To avoid ambiguity associated with thermodynamic efficiency definitions, the previous efficiency-like quantity was replaced by a dimensionless performance indicator (DPI). The relative variation of the DPI is shown in Fig. 5.

**Fig. 6:**
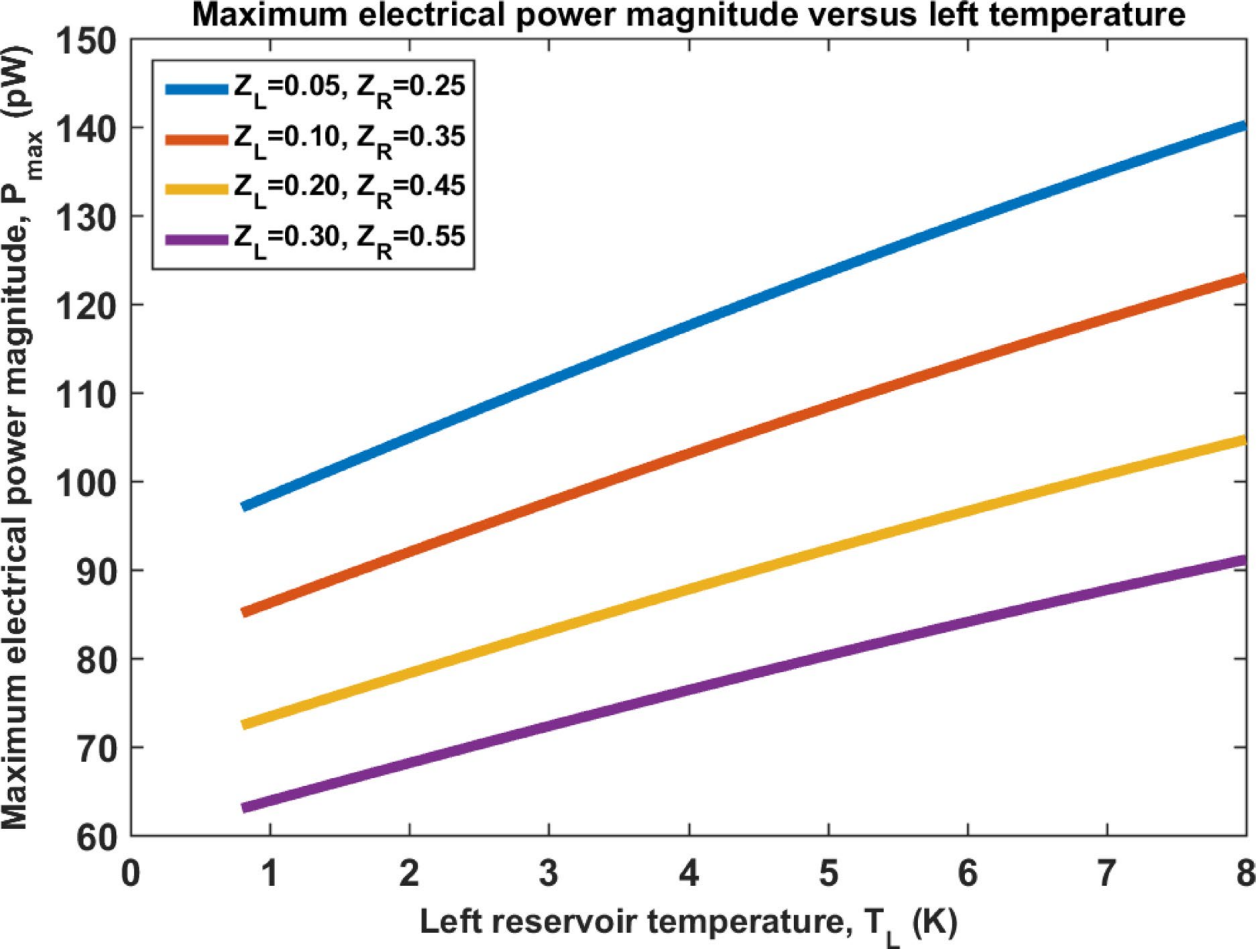
Maximum electrical power magnitude versus left-reservoir temperature.

Only moderate variations are observed under asymmetry modification, indicating that the asymmetry parameters influence both the charge-current magnitude and the heat-current proxy in a partially correlated manner. Consequently, their ratio remains comparatively stable throughout the investigated operating range. The DPI is therefore used only as a comparative transport-performance metric and should not be interpreted as a rigorous thermodynamic efficiency. The influence of the left-reservoir temperature on the maximum electrical power magnitude is illustrated in Fig. 6.

**Fig. 7:**
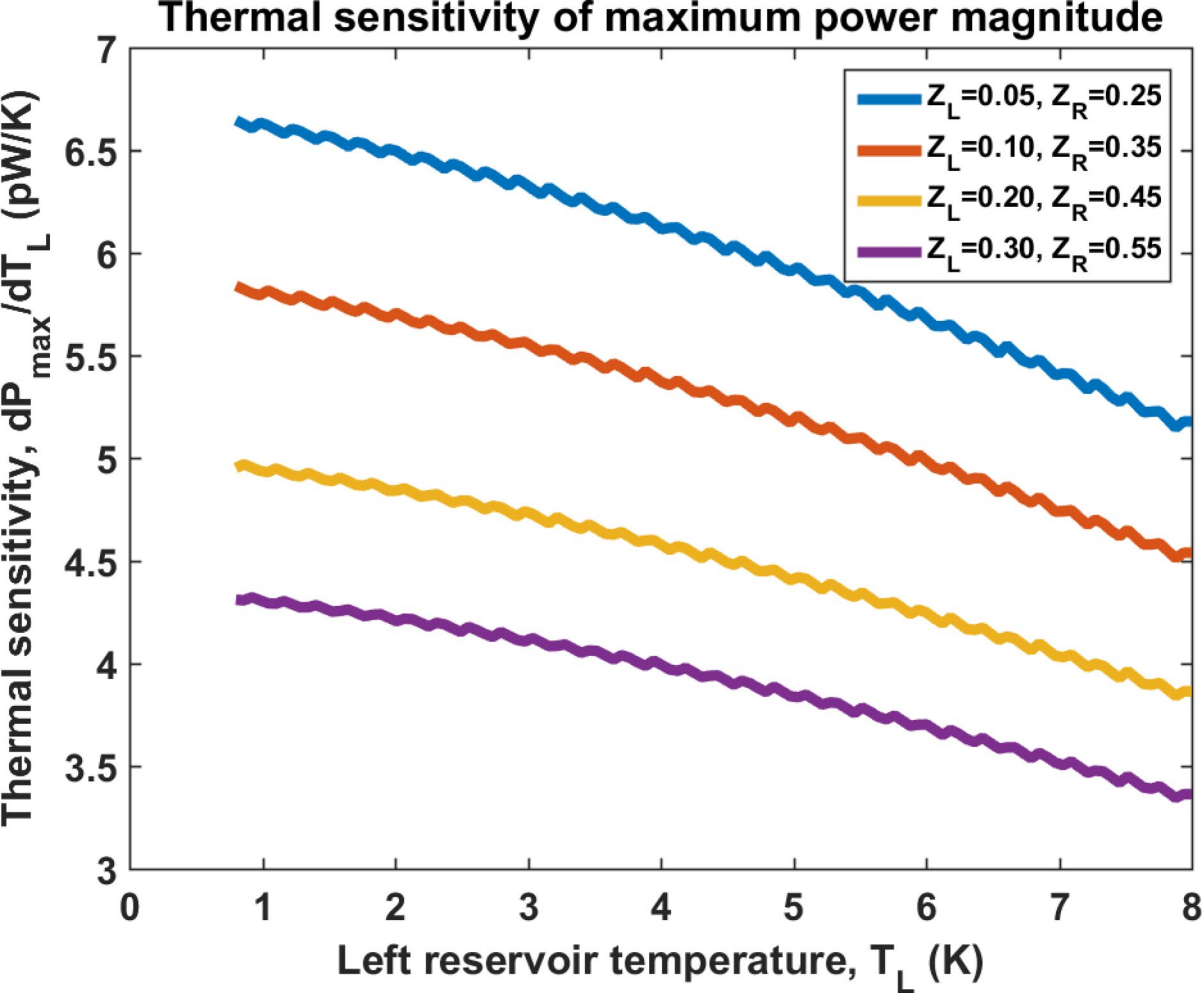
Thermal sensitivity S_T_ = dP_max_/dT_L_ versus left-reservoir temperature.

The maximum power increases approximately linearly with increasing temperature due to the enhancement of the thermal driving force and the corresponding increase in energetically accessible carriers. The observed behavior indicates that thermal modulation plays a significant role in enhancing the transport response of the proposed resonant system. The corresponding thermal sensitivity is presented in Fig. 7.

**Fig. 8:**
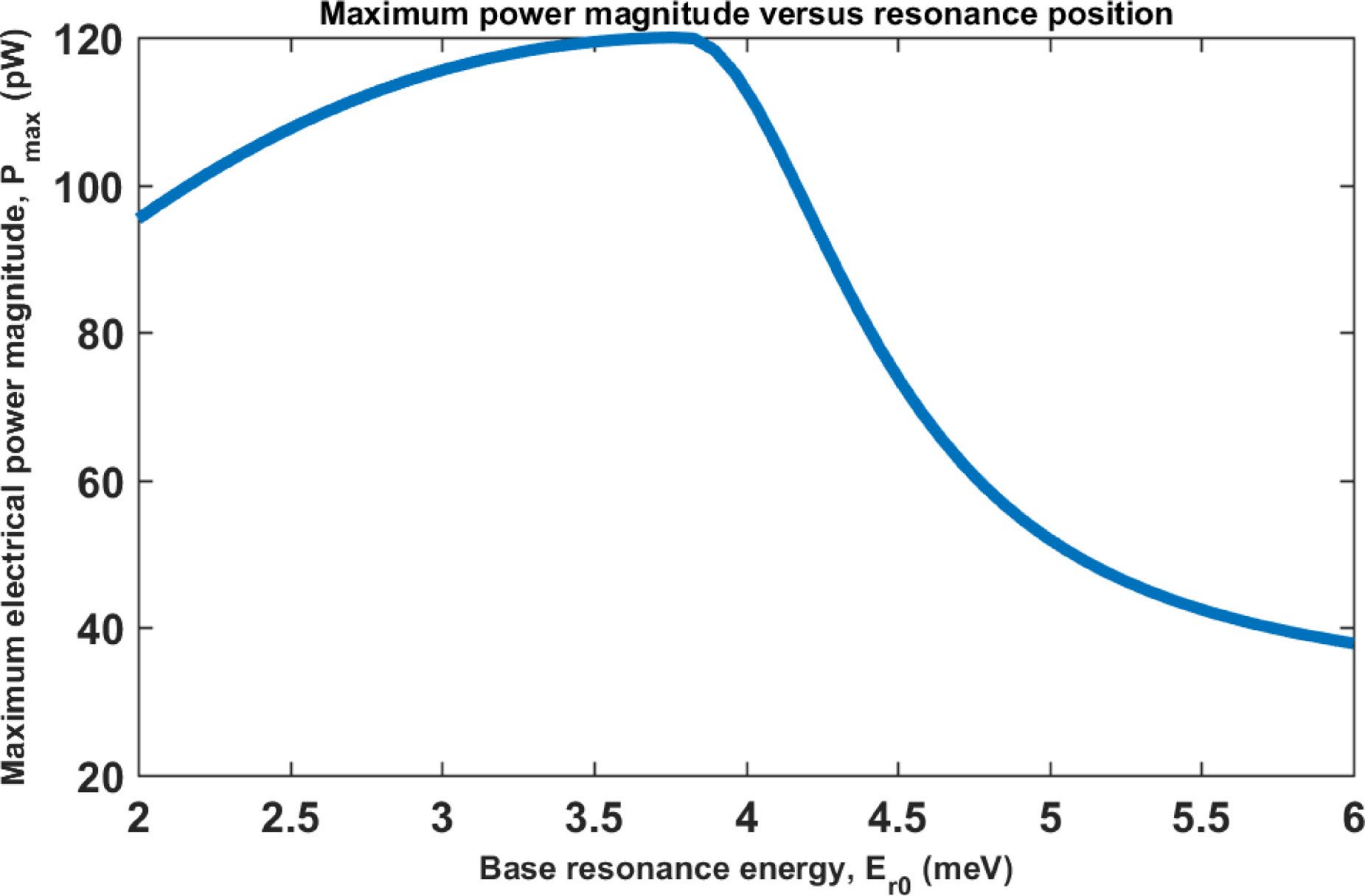
Maximum electrical power magnitude versus base resonance energy E_r0_.

The thermal sensitivity gradually decreases at higher temperatures, suggesting that the system exhibits stronger relative thermal responsiveness at lower operating temperatures. This behavior reflects the competition between increasing transport magnitude and the gradual saturation of the thermal modulation effect included in the phenomenological framework. The dependence of the maximum electrical power magnitude on the resonance energy is shown in Fig. 8.

**Fig. 9:**
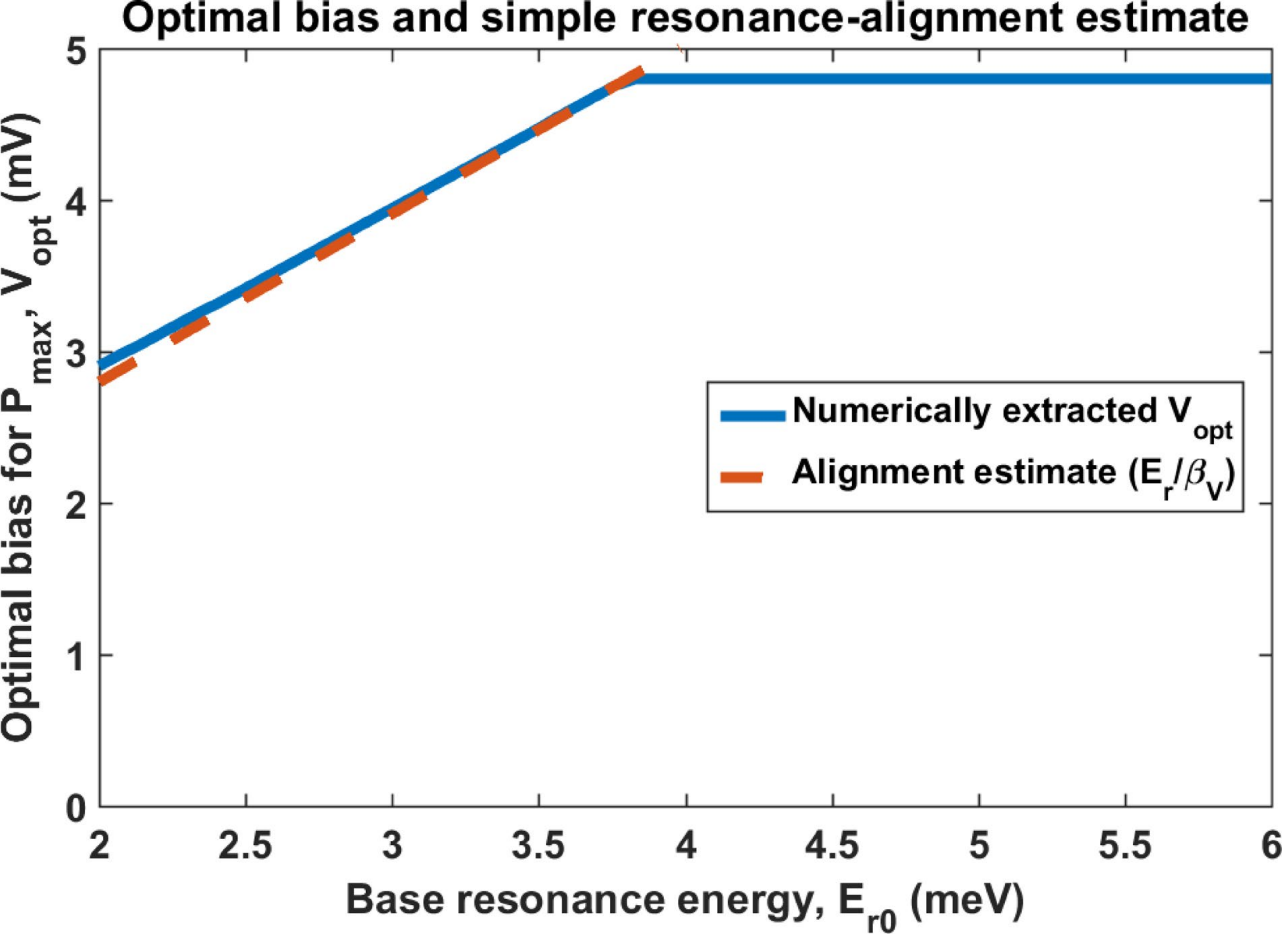
Optimal bias voltage versus base resonance energy E_r0_.

An optimal resonance-energy region is clearly observed due to improved resonance alignment between the effective resonant level and the bias-defined transport window. As the resonance energy approaches the optimal alignment condition, the effective transmission increases and the electrical power magnitude rises accordingly. The variation of the optimal bias voltage with resonance energy is presented in Fig. 9.

**Fig. 10:**
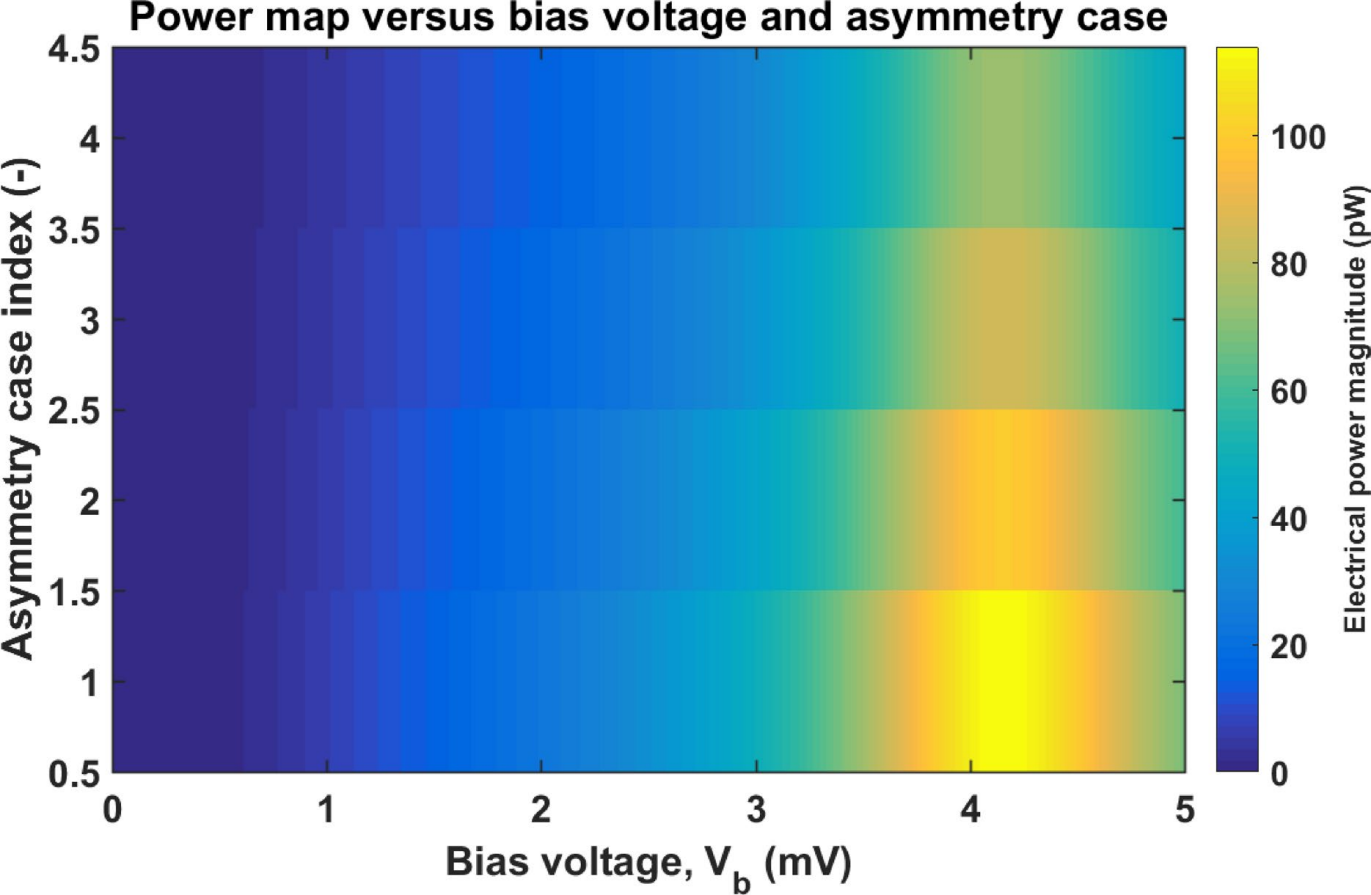
Electrical power map as a function of bias voltage and asymmetry configuration.

The optimal bias voltage increases approximately monotonically with increasing resonance energy, consistent with the resonance-alignment condition included in the effective transport model. At higher resonance energies, the extracted optimal bias exhibits an apparent saturation-like behavior. However, this trend originates primarily from the finite bias-voltage sweep range used in the numerical simulations rather than from a fundamental physical saturation mechanism. A global visualization of the operating regime of the proposed transport system is provided in Fig. 10.

A well-defined high-performance operating region appears near 4–4.5 mV, confirming the existence of a stable resonance-controlled transport regime. Despite variations in structural asymmetry, the dominant operating region remains relatively stable, whereas the transport magnitude decreases systematically with increasing asymmetry. Overall, the obtained results demonstrate that resonance alignment is the primary factor governing the optimal operating condition within the present phenomenological transport framework.

## Discussion and comparison with existing literature

The present results are consistent with the general physical picture established in nanoscale resonant transport, where energy-dependent transmission plays a central role in controlling charge and heat transport. The Landauer framework and its later extensions provide the theoretical basis for describing transport through energy-selective channels, while previous studies on resonant tunneling, molecular thermoelectricity, and quantum-interference-enhanced thermopower have shown that resonance position, transmission shape, and coupling conditions strongly influence thermoelectric response and power output.

Compared with fully microscopic Landauer-based treatments, the present work should be viewed as a compact phenomenological effective model rather than a direct numerical solution of the Landauer integrals. Within this framework, the main contribution is the simultaneous examination of thermal modulation, resonance alignment, finite-bias activation, and structural asymmetry using a computationally efficient formulation. The model therefore provides a physically interpretable way to examine how these effects jointly influence the electrical power magnitude, heat-current proxy, and optimal operating bias. The present results show that increasing structural asymmetry reduces the magnitude of the heat-current proxy, charge-current magnitude, and electrical power magnitude. This trend agrees with the general expectation that weaker or more imbalanced coupling suppresses transport through a resonant junction. However, the optimal bias associated with maximum electrical power magnitude remains only weakly affected by asymmetry in the investigated parameter range. This behavior should not be interpreted as a universal prediction for all nanoscale resonant systems. Rather, it follows from the assumptions of the present phenomenological model, where the asymmetry factor mainly scales the transport amplitude and only weakly modifies the resonance-alignment condition. The resonance-energy sweep further confirms that the optimal operating bias is primarily governed by the alignment between the effective resonance level and the bias-defined transport window. The increase of V_opt_ with E_r0_ supports this interpretation. At higher resonance energies, the apparent flattening of V_opt_ results from the finite bias-sweep range used in the simulations, rather than from a fundamental saturation mechanism. The temperature-dependent results show that the maximum electrical power magnitude increases with the left-reservoir temperature, whereas the thermal sensitivity gradually decreases at higher temperatures. This indicates a trade-off between enhanced power magnitude and reduced relative thermal responsiveness. Such behavior highlights the importance of selecting the operating temperature range according to whether the device is intended mainly for energy-harvesting-type operation or thermal-response evaluation. Overall, the proposed model complements previous microscopic and Landauer-based studies by offering a simplified, reproducible, and physically interpretable framework for studying thermally modulated resonant transport in asymmetric nanoscale junctions. Its main value lies in clarifying trends within a controlled phenomenological setting. However, the model does not include many-body interactions, inelastic scattering, full quantum coherence, phonon heat transport, or a rigorous thermodynamic efficiency calculation. Therefore, future work should validate the present trends using more microscopic transport calculations or experimental calibration where available.

## Conclusion

In this work, a phenomenological effective transport framework was developed to investigate thermally modulated resonant transport in an asymmetric nanoscale junction under coupled electrical and thermal driving conditions. The proposed model combines resonance alignment, thermal modulation, finite-bias activation, damping behavior, and structural asymmetry within a simplified and computationally efficient formulation. Unlike fully microscopic transport treatments, the present approach was designed to provide a physically interpretable description of the dominant transport trends while maintaining numerical simplicity and reproducibility. The obtained results demonstrate that resonance alignment plays the dominant role in determining the transport response and the optimal operating region of the system. Increasing structural asymmetry systematically suppresses the magnitudes of the heat-current proxy, charge-current magnitude, and electrical power magnitude due to weaker effective transport coupling. Nevertheless, the optimal bias associated with maximum electrical power magnitude remains only weakly affected within the investigated parameter range because the asymmetry factor primarily scales the transport amplitude while only weakly modifying the resonance-alignment condition. The temperature-dependent analysis further revealed that the maximum electrical power magnitude increases with increasing thermal driving, whereas the thermal sensitivity gradually decreases at elevated temperatures, indicating a trade-off between transport enhancement and relative thermal responsiveness. In addition, the resonance-energy analysis confirmed that the optimal bias voltage is closely linked to the alignment between the effective resonant level and the bias-defined transport window. The two-dimensional operating map additionally identified a stable high-performance transport regime near 4–4.5 mV. The present study should be interpreted as a phenomenological transport analysis rather than a rigorous microscopic Landauer calculation. Consequently, the model does not include many-body interactions, phonon heat transport, inelastic scattering, full quantum coherence effects, or rigorous thermodynamic efficiency evaluation. Despite these limitations, the framework provides a useful and physically transparent platform for understanding resonance-dominated transport behavior in asymmetric nanoscale systems. Future work may extend the present approach through microscopic quantum transport calculations, experimental calibration, and inclusion of additional non-equilibrium transport mechanisms.

## Data Availability

The datasets used and analyzed in this study are available upon reasonable request from the corresponding author.
